# 1,2,3-Trimeth­oxy-4,5,6-trinitro­benzene

**DOI:** 10.1107/S1600536812004783

**Published:** 2012-02-17

**Authors:** Günter J. Merten, Christian Neis, Kaspar Hegetschweiler

**Affiliations:** a Fachrichtung Chemie, Universität des Saarlandes, Postfach 151150, D-66041 Saarbrücken, Germany

## Abstract

In the title mol­ecule, C_9_H_9_N_3_O_9_, the three neighbouring nitro groups are tilted with respect to the benzene mean plane by 75.8 (1), 27.7 (1) and 68.1 (1)°. The methyl C atoms of the three neighbouring meth­oxy groups deviate from this plane by 0.976 (4), −1.425 (4) and 0.632 (4) Å. The crystal packing exhibits weak C—H⋯O inter­actions.

## Related literature

C—H⋯O hydrogen bonding has been reviewed by Castellano (2004 ▶). The use of aromatic polynitro compounds for the preparation of amino­cyclitols has been reported by Merten *et al.* (2012 ▶). The crystal structures of related highly substituted polynitro benzene derivatives with three meth­oxy or hy­droxy groups in a 1,2,3-arrangement have been reported by Vicente *et al.* (2009 ▶) and Neis *et al.* (2012 ▶), respectively.
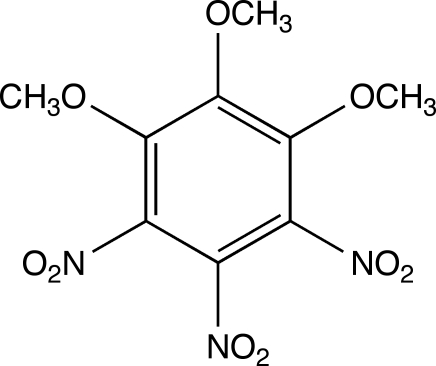


## Experimental

### 

#### Crystal data

C_9_H_9_N_3_O_9_
*M* *_r_* = 303.19
Orthorhombic, 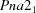

*a* = 8.1743 (4) Å
*b* = 16.6121 (9) Å
*c* = 9.0856 (5) Å
*V* = 1233.75 (11) Å^3^
*Z* = 4
Mo *K*α radiation
μ = 0.15 mm^−1^
*T* = 153 K
0.18 × 0.15 × 0.11 mm

#### Data collection

Bruker APEXII KappaCCD diffractometer
Absorption correction: multi-scan (*SADABS*; Bruker, 2010 ▶) *T*_min_ = 0.974, *T*_max_ = 0.984
10563 measured reflections
1580 independent reflections
1254 reflections with *I* > 2σ(*I*)
*R*_int_ = 0.041

#### Refinement

*R*[*F* ^2^ > 2σ(*F* ^2^)] = 0.034
*wR*(*F* ^2^) = 0.081
*S* = 1.02
1580 reflections
193 parameters
1 restraint
H-atom parameters constrained
Δρ_max_ = 0.16 e Å^−3^
Δρ_min_ = −0.23 e Å^−3^

### 

Data collection: *APEX2* (Bruker, 2010 ▶); cell refinement: *SAINT* (Bruker, 2010 ▶); data reduction: *SAINT*; program(s) used to solve structure: *SHELXS97* (Sheldrick, 2008 ▶); program(s) used to refine structure: *SHELXL97* (Sheldrick, 2008 ▶); molecular graphics: *DIAMOND* (Brandenburg, 2011 ▶); software used to prepare material for publication: *SHELXL97*.

## Supplementary Material

Crystal structure: contains datablock(s) global, I. DOI: 10.1107/S1600536812004783/cv5240sup1.cif

Structure factors: contains datablock(s) I. DOI: 10.1107/S1600536812004783/cv5240Isup2.hkl

Supplementary material file. DOI: 10.1107/S1600536812004783/cv5240Isup3.cml

Additional supplementary materials:  crystallographic information; 3D view; checkCIF report

## Figures and Tables

**Table 1:** Hydrogen-bond geometry (Å, °)

*D*—H⋯*A*	*D*—H	H⋯*A*	*D*⋯*A*	*D*—H⋯*A*
C9—H9*B*⋯O4^i^	0.98	2.53	3.450 (4)	156
C9—H9*C*⋯O9^ii^	0.98	2.59	3.536 (4)	161
C8—H8*A*⋯O5^iii^	0.98	2.44	3.352 (4)	154

Symmetry codes: (i) 
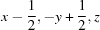
; (ii) 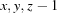; (iii) 
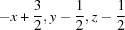
.

**Fig. 1.**
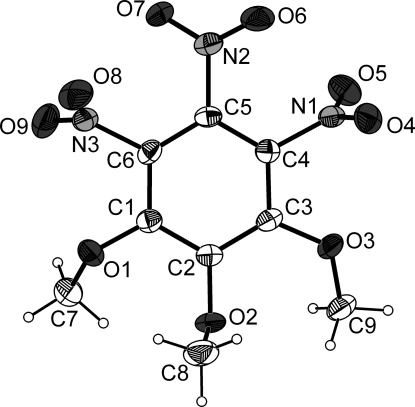
Molecular structure of the title compound. Displacement ellipsoids are drawn at the 50% probability level.

## supplementary crystallographic information

### Comment

Polynitrophenols and their methyl ethers are of interest as possible synthons for the preparation of corresponding aminocyclitols. The crystal structure of the title compound consists of wavy layers which are oriented parallel to the *bc* plane. In these layers, each molecule is surrounded by six neighbours, and the intermolecular contacts within these layers are mainly based on methoxy groups pointing to neighbouring nitro groups, indicating some weak C—H···O—N hydrogen bonding. Between the layers, some of the contacts such as O5···C1 (2.94 Å) are slightly shorter than the sum of the van der Waals radii. Similar to the structure of 4,6-dinitrobenzene-1,2,3-triol, this observation may indicate some weak donor acceptor interactions. However, it should be noted that the tilting of the nitro groups out of the aromatic plane, which is obviously enforced by the increased steric crowding, disfavours a closer approximation of aromatic moieties which are arranged in neighbouring layers.

### Experimental

The title compound was obtained by nitration of 1,2,3-trimethoxybenzene. Caution: 1,2,3-trimethoxy-4,5,6-trinitrobenzene is a potential explosive. ^1^H NMR (CDCl_3_): δ (p.p.m.) = 4.10. ^13^C NMR (CDCl_3_): δ (p.p.m.) = 62.2, 63.4, 130.0, 135.4, 148.1, 151.7. Single crystals were grown by slow evaporation of a MeOH solution at room temperature.

### Refinement

In the absence of significant anomalous scatterers, 932 Friedel pairs were merged before the refinement. H atoms were geometrically positioned (C—H 0.98 Å) and refined as riding, with *U*_iso_(H) = 1.5*U*_eq_ of the pivot atom.

### Crystal data

**Table:**

C_9_H_9_N_3_O_9_	*D*_x_ = 1.632 Mg m^−^^3^
*M**_r_* = 303.19	Mo *K*α radiation, λ = 0.71073 Å
Orthorhombic, *P**n**a*2_1_	Cell parameters from 2358 reflections
*a* = 8.1743 (4) Å	θ = 2.6–22.6°
*b* = 16.6121 (9) Å	µ = 0.15 mm^−^^1^
*c* = 9.0856 (5) Å	*T* = 153 K
*V* = 1233.75 (11) Å^3^	Prism, colourless
*Z* = 4	0.18 × 0.15 × 0.11 mm
*F*(000) = 624	

### Data collection

**Table:**

Bruker APEXII KappaCCD diffractometer	1580 independent reflections
Radiation source: fine-focus sealed tube	1254 reflections with *I* > 2σ(*I*)
Graphite monochromator	*R*_int_ = 0.041
φ and ω scans	θ_max_ = 28.0°, θ_min_ = 2.5°
Absorption correction: multi-scan (*SADABS*; Bruker, 2010)	*h* = −10→7
*T*_min_ = 0.974, *T*_max_ = 0.984	*k* = −17→21
10563 measured reflections	*l* = −12→11

### Refinement

**Table:**

Refinement on *F*^2^	Primary atom site location: structure-invariant direct methods
Least-squares matrix: full	Secondary atom site location: difference Fourier map
*R*[*F*^2^ > 2σ(*F*^2^)] = 0.034	Hydrogen site location: inferred from neighbouring sites
*wR*(*F*^2^) = 0.081	H-atom parameters constrained
*S* = 1.02	*w* = 1/[σ^2^(*F*_o_^2^) + (0.0427*P*)^2^ + 0.1255*P*] where *P* = (*F*_o_^2^ + 2*F*_c_^2^)/3
1580 reflections	(Δ/σ)_max_ < 0.001
193 parameters	Δρ_max_ = 0.16 e Å^−^^3^
1 restraint	Δρ_min_ = −0.23 e Å^−^^3^

### Special details

**Table:**

Geometry. All e.s.d.'s (except the e.s.d. in the dihedral angle between two l.s. planes) are estimated using the full covariance matrix. The cell e.s.d.'s are taken into account individually in the estimation of e.s.d.'s in distances, angles and torsion angles; correlations between e.s.d.'s in cell parameters are only used when they are defined by crystal symmetry. An approximate (isotropic) treatment of cell e.s.d.'s is used for estimating e.s.d.'s involving l.s. planes.
Refinement. Refinement of *F*^2^ against ALL reflections. The weighted *R*-factor *wR* and goodness of fit *S* are based on *F*^2^, conventional *R*-factors *R* are based on *F*, with *F* set to zero for negative *F*^2^. The threshold expression of *F*^2^ > σ(*F*^2^) is used only for calculating *R*-factors(gt) *etc*. and is not relevant to the choice of reflections for refinement. *R*-factors based on *F*^2^ are statistically about twice as large as those based on *F*, and *R*-factors based on ALL data will be even larger.

### Fractional atomic coordinates and isotropic or equivalent isotropic displacement parameters (Å^2^)

**Table:**

	*x*	*y*	*z*	*U*_iso_*/*U*_eq_	
O1	0.7880 (2)	0.02818 (11)	0.3568 (2)	0.0325 (5)	
O2	0.7134 (2)	0.08500 (12)	0.0745 (2)	0.0332 (5)	
O3	0.6929 (3)	0.25609 (12)	0.0347 (2)	0.0355 (5)	
O4	0.8581 (2)	0.38957 (11)	0.1784 (3)	0.0369 (5)	
O5	0.6246 (2)	0.39213 (12)	0.2904 (3)	0.0387 (5)	
O6	0.8947 (3)	0.36515 (13)	0.5039 (2)	0.0442 (6)	
O7	0.8084 (3)	0.27870 (13)	0.6640 (2)	0.0383 (5)	
O8	0.9907 (3)	0.13621 (13)	0.6231 (3)	0.0446 (6)	
O9	0.7445 (3)	0.08999 (15)	0.6539 (2)	0.0485 (6)	
N1	0.7478 (3)	0.35847 (12)	0.2482 (3)	0.0255 (5)	
N2	0.8365 (3)	0.30026 (14)	0.5387 (3)	0.0287 (5)	
N3	0.8482 (3)	0.12674 (13)	0.5858 (3)	0.0315 (5)	
C1	0.7674 (3)	0.10752 (16)	0.3301 (3)	0.0245 (6)	
C2	0.7350 (3)	0.13658 (16)	0.1884 (3)	0.0257 (6)	
C3	0.7256 (3)	0.22000 (17)	0.1639 (3)	0.0261 (5)	
C4	0.7609 (3)	0.27185 (16)	0.2797 (3)	0.0232 (6)	
C5	0.7990 (3)	0.24349 (16)	0.4195 (3)	0.0233 (5)	
C6	0.7997 (3)	0.16148 (17)	0.4432 (3)	0.0248 (6)	
C7	0.6444 (4)	−0.02228 (17)	0.3407 (4)	0.0408 (7)	
H7A	0.5960	−0.0136	0.2433	0.061*	
H7B	0.5643	−0.0084	0.4168	0.061*	
H7C	0.6757	−0.0789	0.3510	0.061*	
C8	0.8624 (4)	0.0513 (2)	0.0168 (4)	0.0471 (9)	
H8A	0.8364	0.0143	−0.0638	0.071*	
H8B	0.9197	0.0221	0.0949	0.071*	
H8C	0.9324	0.0947	−0.0202	0.071*	
C9	0.5854 (4)	0.21928 (19)	−0.0721 (3)	0.0344 (7)	
H9A	0.5431	0.2607	−0.1388	0.052*	
H9B	0.4941	0.1932	−0.0211	0.052*	
H9C	0.6463	0.1790	−0.1288	0.052*	

### Atomic displacement parameters (Å^2^)

**Table:**

	*U*^11^	*U*^22^	*U*^33^	*U*^12^	*U*^13^	*U*^23^
O1	0.0388 (11)	0.0224 (9)	0.0362 (12)	0.0018 (7)	−0.0039 (9)	−0.0010 (9)
O2	0.0433 (11)	0.0329 (11)	0.0235 (11)	−0.0015 (8)	−0.0006 (9)	−0.0110 (9)
O3	0.0506 (11)	0.0346 (11)	0.0212 (10)	−0.0119 (9)	−0.0101 (10)	0.0024 (9)
O4	0.0423 (11)	0.0325 (11)	0.0360 (12)	−0.0070 (9)	0.0115 (10)	0.0034 (9)
O5	0.0322 (10)	0.0330 (10)	0.0508 (14)	0.0058 (8)	0.0067 (10)	−0.0058 (10)
O6	0.0585 (13)	0.0411 (12)	0.0331 (13)	−0.0237 (11)	−0.0018 (11)	−0.0075 (10)
O7	0.0596 (14)	0.0359 (11)	0.0195 (11)	0.0048 (10)	−0.0007 (10)	−0.0029 (9)
O8	0.0416 (12)	0.0522 (13)	0.0401 (13)	0.0123 (10)	−0.0188 (10)	−0.0059 (10)
O9	0.0640 (15)	0.0502 (13)	0.0312 (13)	−0.0101 (11)	−0.0018 (11)	0.0120 (11)
N1	0.0282 (11)	0.0268 (12)	0.0216 (11)	−0.0023 (9)	0.0006 (10)	−0.0046 (11)
N2	0.0285 (11)	0.0332 (13)	0.0245 (14)	0.0013 (9)	−0.0035 (10)	−0.0063 (10)
N3	0.0431 (14)	0.0274 (12)	0.0240 (13)	0.0073 (10)	−0.0068 (11)	−0.0033 (10)
C1	0.0227 (12)	0.0268 (13)	0.0240 (15)	0.0017 (9)	0.0008 (11)	−0.0022 (11)
C2	0.0274 (14)	0.0264 (13)	0.0234 (14)	−0.0009 (10)	0.0003 (11)	−0.0052 (11)
C3	0.0268 (13)	0.0326 (13)	0.0188 (13)	−0.0028 (11)	−0.0012 (11)	−0.0023 (11)
C4	0.0216 (12)	0.0248 (13)	0.0232 (14)	−0.0018 (10)	0.0024 (11)	−0.0009 (11)
C5	0.0204 (11)	0.0276 (14)	0.0218 (14)	0.0013 (10)	0.0009 (10)	−0.0066 (11)
C6	0.0240 (13)	0.0306 (13)	0.0198 (14)	0.0041 (10)	−0.0027 (11)	0.0007 (11)
C7	0.0532 (19)	0.0317 (15)	0.0376 (17)	−0.0101 (12)	−0.0055 (16)	0.0010 (15)
C8	0.061 (2)	0.0464 (19)	0.0341 (19)	0.0180 (16)	−0.0004 (15)	−0.0148 (15)
C9	0.0383 (16)	0.0441 (17)	0.0209 (14)	−0.0062 (12)	−0.0039 (12)	−0.0013 (13)

### Geometric parameters (Å, º)

**Table:**

O1—C1	1.351 (3)	C1—C6	1.389 (4)
O1—C7	1.451 (3)	C1—C2	1.400 (4)
O2—C2	1.355 (3)	C2—C3	1.406 (4)
O2—C8	1.439 (4)	C3—C4	1.390 (4)
O3—C3	1.345 (3)	C4—C5	1.390 (4)
O3—C9	1.445 (3)	C5—C6	1.379 (4)
O4—N1	1.218 (3)	C7—H7A	0.9800
O5—N1	1.214 (3)	C7—H7B	0.9800
O6—N2	1.220 (3)	C7—H7C	0.9800
O7—N2	1.215 (3)	C8—H8A	0.9800
O8—N3	1.224 (3)	C8—H8B	0.9800
O9—N3	1.214 (3)	C8—H8C	0.9800
N1—C4	1.471 (3)	C9—H9A	0.9800
N2—C5	1.469 (3)	C9—H9B	0.9800
N3—C6	1.472 (4)	C9—H9C	0.9800
			
C1—O1—C7	116.4 (2)	C6—C5—C4	118.6 (2)
C2—O2—C8	114.4 (2)	C6—C5—N2	121.2 (2)
C3—O3—C9	121.2 (2)	C4—C5—N2	120.2 (2)
O5—N1—O4	125.7 (2)	C5—C6—C1	121.4 (2)
O5—N1—C4	116.7 (2)	C5—C6—N3	121.7 (2)
O4—N1—C4	117.5 (2)	C1—C6—N3	116.7 (2)
O7—N2—O6	125.2 (2)	O1—C7—H7A	109.5
O7—N2—C5	117.5 (2)	O1—C7—H7B	109.5
O6—N2—C5	117.2 (2)	H7A—C7—H7B	109.5
O9—N3—O8	126.0 (3)	O1—C7—H7C	109.5
O9—N3—C6	117.2 (2)	H7A—C7—H7C	109.5
O8—N3—C6	116.7 (2)	H7B—C7—H7C	109.5
O1—C1—C6	118.2 (2)	O2—C8—H8A	109.5
O1—C1—C2	121.7 (2)	O2—C8—H8B	109.5
C6—C1—C2	119.6 (2)	H8A—C8—H8B	109.5
O2—C2—C1	120.6 (2)	O2—C8—H8C	109.5
O2—C2—C3	119.7 (2)	H8A—C8—H8C	109.5
C1—C2—C3	119.7 (2)	H8B—C8—H8C	109.5
O3—C3—C4	115.2 (2)	O3—C9—H9A	109.5
O3—C3—C2	126.1 (3)	O3—C9—H9B	109.5
C4—C3—C2	118.7 (2)	H9A—C9—H9B	109.5
C3—C4—C5	121.9 (2)	O3—C9—H9C	109.5
C3—C4—N1	116.4 (2)	H9A—C9—H9C	109.5
C5—C4—N1	121.7 (2)	H9B—C9—H9C	109.5
			
C7—O1—C1—C6	119.7 (3)	O4—N1—C4—C5	106.2 (3)
C7—O1—C1—C2	−68.4 (3)	C3—C4—C5—C6	−0.7 (4)
C8—O2—C2—C1	−77.5 (3)	N1—C4—C5—C6	176.2 (2)
C8—O2—C2—C3	102.2 (3)	C3—C4—C5—N2	179.7 (2)
O1—C1—C2—O2	4.1 (4)	N1—C4—C5—N2	−3.5 (4)
C6—C1—C2—O2	175.8 (2)	O7—N2—C5—C6	−26.9 (4)
O1—C1—C2—C3	−175.6 (2)	O6—N2—C5—C6	152.5 (3)
C6—C1—C2—C3	−3.9 (4)	O7—N2—C5—C4	152.7 (2)
C9—O3—C3—C4	−151.0 (2)	O6—N2—C5—C4	−27.9 (3)
C9—O3—C3—C2	32.6 (4)	C4—C5—C6—C1	1.6 (4)
O2—C2—C3—O3	1.4 (4)	N2—C5—C6—C1	−178.7 (2)
C1—C2—C3—O3	−178.9 (2)	C4—C5—C6—N3	176.3 (2)
O2—C2—C3—C4	−175.0 (2)	N2—C5—C6—N3	−4.0 (4)
C1—C2—C3—C4	4.8 (4)	O1—C1—C6—C5	172.7 (2)
O3—C3—C4—C5	−179.3 (2)	C2—C1—C6—C5	0.7 (4)
C2—C3—C4—C5	−2.5 (4)	O1—C1—C6—N3	−2.3 (3)
O3—C3—C4—N1	3.7 (3)	C2—C1—C6—N3	−174.3 (2)
C2—C3—C4—N1	−179.5 (2)	O9—N3—C6—C5	116.1 (3)
O5—N1—C4—C3	101.0 (3)	O8—N3—C6—C5	−65.4 (3)
O4—N1—C4—C3	−76.8 (3)	O9—N3—C6—C1	−69.0 (3)
O5—N1—C4—C5	−76.0 (3)	O8—N3—C6—C1	109.5 (3)

### Hydrogen-bond geometry (Å, º)

**Table:**

*D*—H···*A*	*D*—H	H···*A*	*D*···*A*	*D*—H···*A*
C9—H9*B*···O4^i^	0.98	2.53	3.450 (4)	156
C9—H9*C*···O9^ii^	0.98	2.59	3.536 (4)	161
C8—H8*A*···O5^iii^	0.98	2.44	3.352 (4)	154

Symmetry codes: (i) *x*−1/2, −*y*+1/2, *z*; (ii) *x*, *y*, *z*−1; (iii) −*x*+3/2, *y*−1/2, *z*−1/2.
